# Supplementary material for: Evaluating the Energy and Core Nutrients of Condiments in China
Source: Nutrients. 2023 Oct 12;15(20):4346. doi: 10.3390/nu15204346 (PMC10609505; doi:10.3390/nu15204346)
Supplement: Supplementary file 1 [file nutrients-15-04346-s001.zip › nutrients-2575076-Supplementary files.pdf]

Supplementary Table S1: Detailed description of data collection for each country.

Country	Data source	Year/s of data collection	Data collection description
**Belgium**	**National Open Database**	**2009-2022**	VoG Nubel (Nutriënten België) was founded on 9 March 1990, with a revised statute published in the Belgian Official Gazette on 13 July 2009. The objective of organisation is to help Belgians obtain the correct scientific information so that they can compose their diet in a balanced way. The database provides a wealth of nutrient knowledge to help people make conscious dietary choices and maintain a healthy lifestyle based on a balanced diet. The VoG Nubel is made up of partners from the public sector, the Federal Public Service Public Health, Food Chain Safety & Environment and the Scientific Institute of Public Health (WIV-ISP).<br>This website is an interactive brand database where detailed information on each food item on the Belgian market can be found. In addition to this, it is possible to consult the nutritional value of no less than 7,000 food products - 6,000 of which are branded products - (the content of energy, fat, sodium and other nutrients), as well as the standardization of weights and measures.<br>Both nutrition professionals and consumers can find out accurate information about the nutritional components of different branded products and basic foods here.<br>Please cite as:<br>https://www.internubel.be/
**UK**	**National Open Database**	**2021**	The 2021 labelling dataset covers 2886 foods and ingredients from almonds and apples to yogurt and Yorkshire puddings, which contains reference data on the levels of nutrients in thousands of the most commonly eaten foods in the UK.These values were taken from the 2019 Food Composition Comprehensive Dataset and recalculated into the format required for nutrition labelling. Foods considered to be ingredients have been filled in with the best knowledge and information available to fill in the gaps.This includes many sources, such as manufacturers' data, scientific literature, food composition datasets from other countries and calculations based on similar foods and ingredients. This new labelling new dataset will help food manufacturers, dieticians,

			nutritionists, software and app producers create the nutrition information for food products and recipes. The 2021 dataset will make the use of the UK food composition dataset far easier for food labelling purposes.<br>Please cite as:<br>UK Composition of Foods Labelling dataset 2021, H Pinchen1, L Zhang1, M Roe2, S Church3, M Traka1, P Finglas1; 1Quadram Institute Bioscience, 2EuroFIR AISBL, 3Independent Nutritionist, April 2021
**France**	**National Open Database**	**2019**	Oqali, formerly known as the « Food Quality Observatory », was introduced as part of the second National Nutrition and Health Program (PNNS 2).The Oqali database brings together much of the information labelled on the packaging of processed food products on the French market (descriptive characteristics of the product, nutritional values, lists of ingredients, etc.). It is not exhaustive. Data is collected from a number of sources: mainly packaging sent in by food industry professionals (manufacturers, distributors, etc.), photos taken in shops and, occasionally, product purchases. It is structured by food category, each of them being regularly collected in order to follow the evolution of the food offer over time (thus, all food categories are not collected the same year and the information made available corresponds to the products available on the market at a given time).<br>Please cite as:<br>https://www.oqali.fr/donnees-publiques/base-de-donnees-oqali/.
**Japan**	**National Open Database**	**2015**	The most recent version of the food composition table for Japan was published by the Ministry of Education, Culture, Sports, Science and Technology and the latest version of the Standard Tables of Food Composition in Japan-2015-comprises the main food composition table (Standard Tables of Food Composition in Japan-2015-(Seventh revised Edition)) and three supplementary books. The supplementary books are Standard Tables of Food Composition in Japan - 2015 - (Seventh Revised Edition) - Amino Acids -, Standard Tables of Food Composition in Japan - 2015 - (Seventh Revised Edition) - Fatty Acids - and Standard Tables of Food Composition in Japan - 2015 - (Seventh Revised Edition) - Available Carbohydrates, Polyols and Organic Acids-. This food composition table is expected to play an important role as one of the main tables of the East Asia food composition tables.<br>Please cite as:<br>https://www.mext.go.jp/en/policy/science_technology/policy/title01/detail01/1374030.htm

Supplementary Table S2: Collect the various categories and quantities of foreign related condiments

Country	Food category	Subcategory	Preliminary collection	Remaining after filtering
			n	n
UK			127	99
	Mayonnaise/ Salad dressing		26	20
	Tomato ketchup		6	6
	Bouillon cubes		14	13
	Soup, not concentrated		25	25
	Compound seasoning		56	35
France			1119	1035
	Mayonnaise/ Salad dressing		197	172
	Tomato ketchup		338	327
	Compound seasoning		584	536
		Sauces bolognaises et assimiles	221	212
		Sauces condimentaires_RHF	125	91
		Sauces pour poisson	36	33
		Vinaigrettes allegees en matieres grasses	202	200
Belgium			391	386
	Soy sauces		18	18
	Mayonnaise/ Salad dressing		121	121
	Tomato ketchup		17	17
	Compound seasoning		230	230
	Other condiments category		5	0
Japan			129	113
	Mayonnaise/ Salad dressing		5	5
	Tomato ketchup		5	5
	Spicy agent		28	28
	Soy sauces		14	14
	Vinegar		6	6
	Salt		4	4
	Soybean Sauce		11	11

Bouillon cubes	6	6
Compound seasoning	34	34
Other condiments category	16	0
Total	1766	1633

Supplementary Table S3: Food category standard definitions

	Food category	Definitions
1	Vinegar	A liquid condiment made by microbial fermentation of various materials containing starch, sugar or alcohol, alone or in combination.
2	Soy sauces	Liquid condiments with special colour, flavour and taste made by microbial fermentation using soybeans and/or defatted soybeans, wheat and/or wheat flour and/or bran as the main raw materials, including brewing soy sauce, prepared soy sauce, etc.
3	Fermented bean curd	A seasoning and meal preparation product made from soybeans by processing, grinding, baking, cultivating and fermenting.
4	Sugar/sweeteners	Sugar used for flavouring generally refers to white granulated sugar or sheep sugar refined from sugar cane or sugar beet, but also includes starch syrup, caramel, glucose, lactose, etc.
5	Salt	Also known as table salt. Salt used for cooking, seasoning and curing, with sodium chloride as the main ingredient. According to its production and processing methods it can be divided into refined salt, crushed and washed salt and sun-dried salt.
6	Spicy agent（cayenne pepper、sprinkling pepper）	Products made from a variety of spices, with or without the addition of auxiliary ingredients. The spices are mainly from the fruits, stems, leaves, bark and roots of various naturally growing plants and have a strong aromatic, pungent flavour.
7	Pickled vegetable	A variety of vegetable products made from fresh vegetables, such as pickled vegetables, salt pickled vegetables, soy sauce pickled vegetables, sugar pickled vegetables, vinegar pickled vegetables, sweet and sour pickled vegetables, shrimp oil pickled vegetables, fermented pickled vegetables and bad pickled vegetables, etc.
8	Paste and like products	These include sesame, bean and sweet noodle sauces as well as mushroom and beef sauces in a variety of flavours.<br>Chinese bean paste: A sauce made from soya beans by microbial fermentation.<br>Sesame paste/Peanut butter:<br>Sesame paste: Also known as sesame sauce. The sauce made of sesame seeds is made by moistening, hulling,

		roasting and grinding, and some add other auxiliary ingredients.<br>Peanut butter: Peanut fruits are shelled and decoated, and then roasted and ground to make sauces, and some add other auxiliary ingredients.
9	Chicken essence	A compound seasoning made from MSG, edible salt, powdered chicken meat or bones or their concentrated extracts, disodium nucleotide and other auxiliary ingredients, with or without the addition of flavour enhancers such as spices and/or edible flavourings, which are mixed and dried to give the freshness and flavour of chicken.
10	Bouillon cubes	A compound flavouring made from plants and animals and/or their concentrated extracts as the main flavouring raw material, with salt and other seasonings and auxiliaries, dried and processed.
11	Mayonnaise/ Salad dressing	Mayonnaise: Western-style condiment. A sour semi-solid emulsified sauce made from vegetable oil, acidic ingredients (vinegar, acidifier) and egg yolk, supplemented by modified starch, sweetener, salt, spices, emulsifiers and thickeners, mixed and homogenised.<br>Salad dressing: Western-style condiment. A sour semi-solid emulsified sauce made from vegetable oil, acidic ingredients (vinegar, acidifier), supplemented by modified starch, sweetener, salt, spices, emulsifiers and thickeners, mixed and homogenised.
12	Tomato ketchup	Sauces made with tomatoes (tomatoes) as raw materials, with or without salt, sugar and food additives, and varieties with added auxiliary materials can be called tomato sauce.
13	Oyster sauces/Fish sauces	Oyster sauces: Use oyster steamed and boiled juice to concentrate or directly use oyster meat enzymatic hydrolysis, and then add sugar, salt, starch or modified starch and other raw materials, supplemented by other ingredients and food additives made of condiments.<br>Fish sauces: Umami liquid condiments made from fish, shrimp and shellfish under biological enzymatic hydrolysis under high salt.
14	Hot pot seasoning	Seasonings used to eat hot pot, including hot pot base and hot pot dipping.<br>Hot pot base: Seasonings made from animal and vegetable fats, chillies, sugar, salt, monosodium glutamate, spices, bean paste, etc., processed according to a certain formula and process and used to make hot pot soup.<br>Hot pot Dipping Sauce: It is made of sesame paste, beancurd, leek flower, pepper, salt, monosodium glutamate and other condiments and used for dipping in hot pot.

15	Compound seasoning	With two or more kinds of seasonings as raw materials, adding or not adding auxiliary materials, processed by the corresponding process can be liquid, semi-solid or solid products.<br>Include: Other solid compound seasoning, Sauce based on vegetable ingredients, Sauce based on animal ingredients, Other semi-solid compound sauces, Seasoned Green Seasoning

Note: The food category standard definition comes from China's domestic relevant standards, respectively “GB 2760-2014”，“GB/T 20903-2007”，“GB/T 24399-2009”，“GB 2717-2018”，“GB 2714-2015”.
